# Immunoglobulin G4-related constrictive pericarditis identified by cytological examination of pericardial effusion: a case report

**DOI:** 10.1186/s13256-016-1159-1

**Published:** 2016-12-20

**Authors:** Kazunori Horie, Norio Tada, Keiichirou Yamaguchi, Keitarou Inazawa, Mareyuki Endo, Naoto Inoue

**Affiliations:** 1 Department of Cardiovascular Medicine, Sendai Kousei Hospital, Sendai, Miyagi Japan; 2 Department of Radiology, Sendai Kousei Hospital, Sendai, Miyagi Japan; 3 Department of Respiratory Surgery, Sendai Kousei Hospital, Sendai, Miyagi Japan; 4 Department of Pathology, Sendai Kousei Hospital, Sendai, Miyagi Japan; 5 Division of Cardiovascular Medicine, Sendai Kousei Hospital, 4-15 Hirose-cho, Aoba-ku, Sendai, Miyagi 980-0873 Japan

**Keywords:** IgG4-related disease, Cytological examination, Constrictive pericarditis, Positron-emission tomography, Case report

## Abstract

**Background:**

Immunoglobulin G4-related disease is increasingly recognized as a systemic autoimmune disorder characterized by immunoglobulin G4-positive lymphocyte infiltration. Organ biopsy and histopathology are the most important diagnostic methods; however, the significance of a cytological examination in immunoglobulin G4-related disease cases is still unclear.

**Case presentation:**

A 73-year-old Asian man who was a former tobacco smoker presented with progressive exertional dyspnea, systemic edema, and pericardial effusion. A cytological examination of his pericardial effusion detected three or four plasma cells per high-power field by Giemsa staining. Moreover, immunoglobulin G4-positive plasma cells were detected by immunostaining. Cardiac catheterization after pericardiocentesis revealed that both ventricular pressure traces showed an early diastolic dip and plateau. Positron-emission tomography with ^18^F-fluorodeoxyglucose imaging revealed inflammatory foci in his pericardium. A surgical pericardiectomy was performed and the resultant specimen showed significant immunoglobulin G4-positive plasma cell infiltration and marked fibrous thickening of his pericardium; therefore, a diagnosis of constrictive pericarditis due to immunoglobulin G4-related disease was made. Oral administration of 0.6-mg/kg/day prednisolone resolved his heart failure and he was discharged on foot 1 week later.

**Conclusion:**

Our experience with this case indicates that cytological examination of pericardial effusion was useful in the diagnosis of immunoglobulin G4-related disease.

## Background

Immunoglobulin G4 (IgG4)-related disease (IgG4-RD) is a systemic inflammatory disease characterized by IgG4-positive lymphocyte infiltration that causes fibrosclerotic change in various tissues and organs [1, 2]. Although the diagnostic criteria for IgG4-RD include histopathological findings in a biopsy specimen [2], the significance of a cytological examination is still unknown. Here, we describe the case of a patient with IgG4-RD who presented with constrictive pericarditis (CP) that was identified by IgG4-positive plasma cells in pericardial effusion and was confirmed by a surgical pericardiectomy.

## Case presentation

**Fig. 1:**
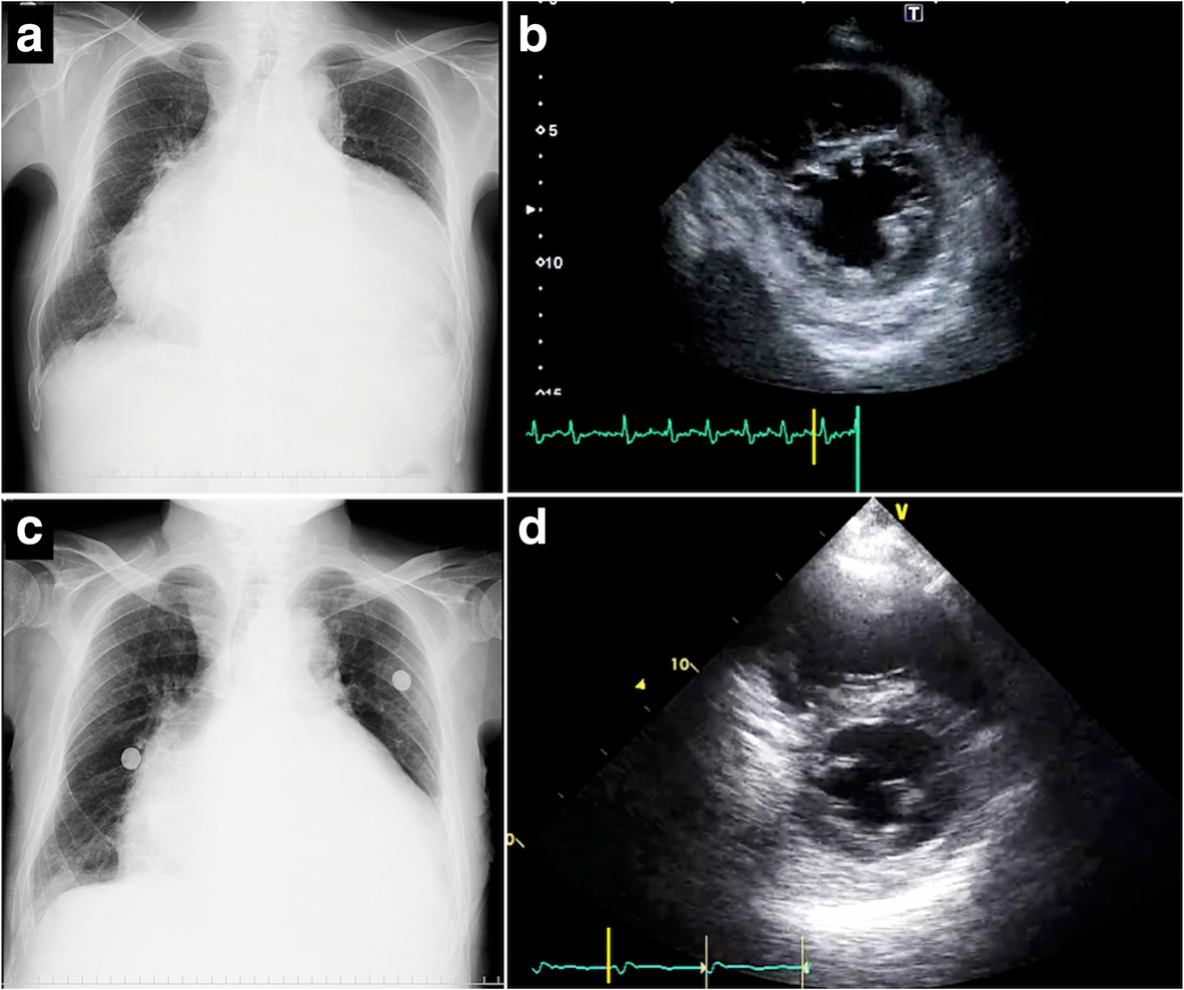
The findings of chest X-ray and transthoracic echocardiography during hospitalization. **a** The chest X-ray on the first day of hospitalization showed an increased cardiothoracic ratio of 84.4% and bilateral pleural effusion. **b** The end-diastolic ventricular septal shift was still present after removal of the pericardial effusion, as evaluated by transthoracic echocardiography. **c** A chest X-ray after the administration of oral corticosteroid therapy detected a reduced cardiothoracic ratio of 73.4%. **d** Transthoracic echocardiography after the administration of oral corticosteroid therapy detected that the diastolic ventricular septal shift was improved at discharge

A 73-year-old Asian man, a former tobacco smoker with hypertension and diabetes, presented to the emergency department in our hospital with a 2-month history of progressive exertional dyspnea. He was diagnosed with congestive heart failure due to arterial fibrillation and tricuspid regurgitation; he had been hospitalized five times over the previous 5 years and had been treated with bisoprolol and furosemide. Pericardial friction rub or knock, or pericardial effusion was not detected in any previous hospitalizations. He had a family history of congestive heart failure, lung cancer, and gallbladder cancer. He was prescribed 2.5 mg bisoprolol, 40 mg furosemide, 60 mg azosemide, and 80 mg valsartan before the current illness. An initial physical examination on the first day of hospitalization revealed the following: blood pressure, 101/56 mmHg; pulse rate, 108 beats/minute; respiratory rate, 20 breaths/minute; body temperature, 37.0 °C; and oxygen saturation 95% while he was breathing room air. Jugular venous distension, Kussmaul’s sign, and leg edema were observed. A neurological examination did not reveal any abnormal objective findings. Chest radiography revealed bilateral pleural effusion with an increased cardiothoracic ratio of 84.4% (Fig. 1a). Laboratory tests indicated that his serum levels of immunoglobulin G (IgG) (1729 mg/dL) and its subclass IgG4 (122.0 mg/dL) were elevated. His serum levels of triiodothyronine, thyroxine, and thyroid-stimulating hormone were all within normal limits. He was negative for an antinuclear antibody, an anti-deoxyribonucleic acid enzyme-linked immunosorbent assay, p-antineutrophil or c-antineutrophil cytoplasmic antibodies, and a lupus anticoagulant. Sputum acid-fast bacillus cultures and the tuberculin test were also negative.

**Fig. 2:**
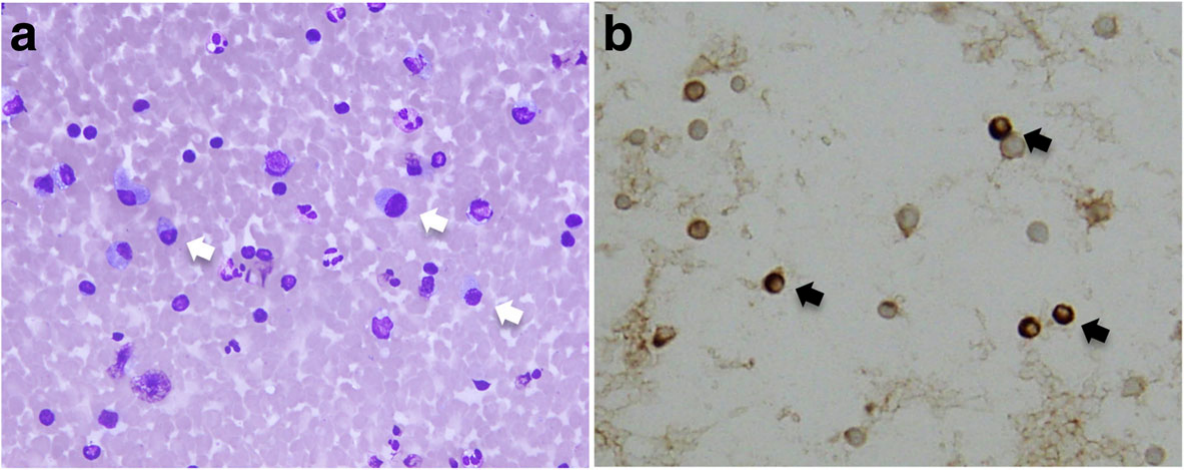
The findings from cytological examination of the pericardial effusion. **a** Giemsa staining revealed three or four plasma cells per high-power field in the pericardial effusion (*white arrows*). **b** Immunoglobulin G4-positive plasma cells were detected in the pericardial effusion by immunostaining (*black arrows*)

**Fig. 3:**
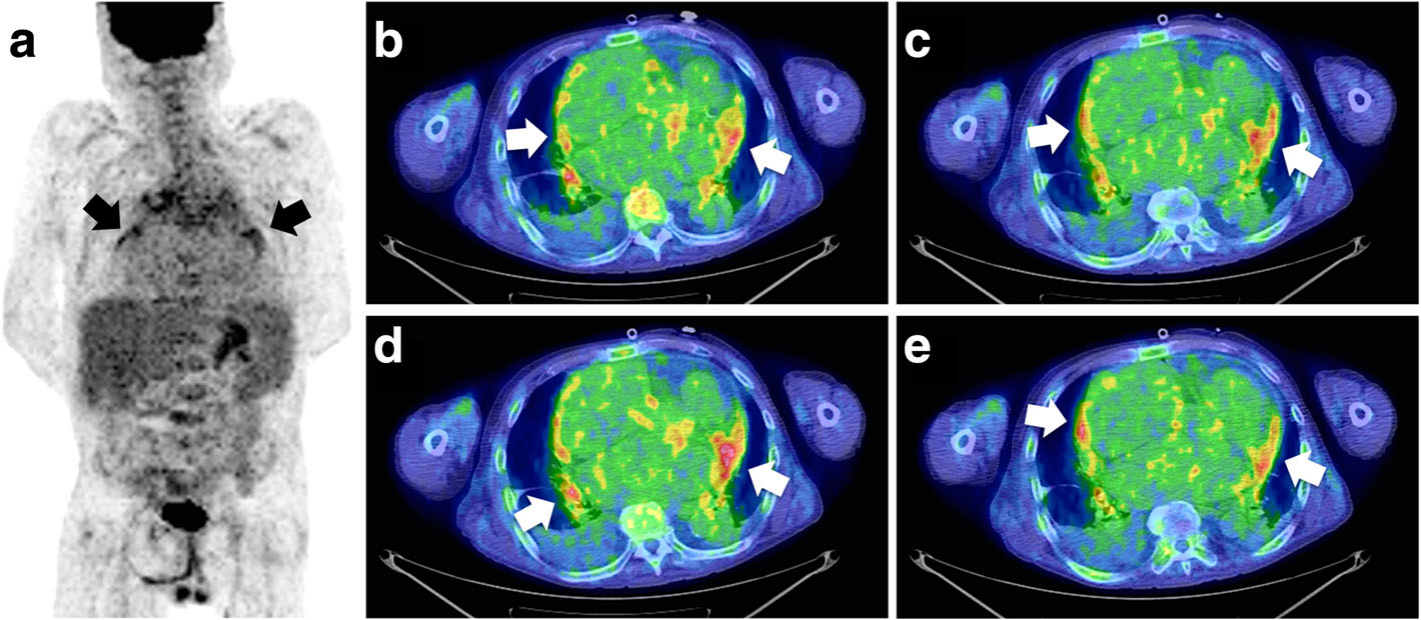
The inflammatory foci in the pericardium as detected by positron emission tomography with ^18^F-fluorodeoxyglucose. **a** Positron emission tomography imaging detected localized uptake of ^18^F-fluorodeoxyglucose in the pericardium (*black arrows*). **b** to **e** Serial horizontal cross-sectional images demonstrating the accumulation of ^18^F- fluorodeoxyglucose in both sides of the pericardium (*white arrows*)

Transthoracic echocardiography (TTE) demonstrated pericardial effusion with a pericardial cavity that was 24-mm thick. Pericardiocentesis revealed 900 mL of exudative effusion, Giemsa staining revealed three or four plasma cells per high-power field in the pericardial effusion (Fig. 2a), and IgG4-positive plasma cells were detected by immunostaining (Fig. 2b). Even after pericardial drainage, his symptoms persisted and TTE showed an end-diastolic ventricular septal shift (Fig. 1b). Cardiac catheterization revealed that both ventricular pressure traces showed an early diastolic dip and plateau. Moreover, significant reductions in both ventricular peak systolic pressures during inspiration were observed. Although intravenous furosemide and dobutamine infusion in addition to 15.0 mg of oral tolvaptan were prescribed, his symptoms were not resolved. Positron-emission tomography (PET) imaging detected an abnormal uptake of ^18^F-fluorodeoxyglucose (^18^F-FDG) in his pericardium as well as in his gastric wall and in his hilar lymph nodes (Fig. 3a). Serial horizontal cross-sectional images demonstrated ^18^F-FDG uptake in both sides of his pericardium (Fig. 3b–e).

**Fig. 4:**
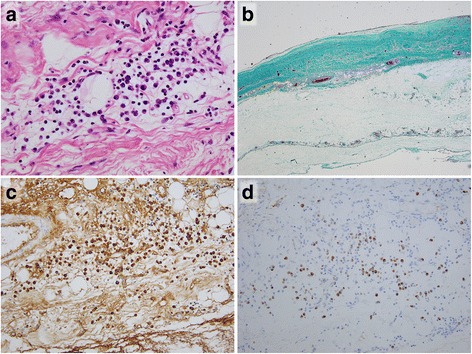
The histopathological appearance of the pericardium. **a** A hematoxylin and eosin-stained section of the pericardium showed lymphoplasmacytic inflammation with scattered plasma cells among a fibrous stroma (original magnification, ×200). **b** The elastica Masson–Goldner-stained section showed marked fibrous thickening of the pericardium extending into the fatty tissue (original magnification, ×40). **c** and **d** Formalin-fixed, paraffin-embedded tissue with immunostaining directed against immunoglobulin G (**c**) and immunoglobulin G4 (**d**); immunoglobulin G and immunoglobulin G4 staining, original magnifications, ×200. The ratio of immunoglobulin G4-positive plasma cells/immunoglobulin G-positive plasma cells was 42%

A thoracoscopic pericardiectomy was performed and a histopathological analysis demonstrated lymphoplasmacytic inflammation with scattered plasma cells among a fibrous stroma in specimens of the pericardium in hematoxylin and eosin-stained sections (Fig. 4a). Elastica Masson–Goldner-stained sections revealed fibrous thickening of the pericardium (Fig. 4b). Immunostaining showed an IgG4/IgG-positive plasma cell ratio of 42% (Fig. 4c and d). Although our patient’s serum IgG4 level did not reach the diagnostic criterion of >135.0 mg/dL, he was diagnosed as having IgG4-RD because of typical histopathological features and the clinical symptoms of CP [2]. He was administered 30 mg of oral prednisolone (0.6 mg/kg/day) for 2 weeks and the dose was gradually decreased over the following 2 months to a maintenance dose of 2.5 to 5.0 mg daily [2]. Soon after starting on prednisolone, his dyspnea and leg edema dramatically improved over a 1-week period. An X-ray showed the resolution of bilateral pleural effusion and a decreased cardiothoracic ratio to 73.4% (Fig. 1c). Moreover, TTE revealed that the end-diastolic ventricular septal shift had disappeared (Fig. 1d). He was discharged on foot 1 week after he had been introduced to corticosteroid therapy.

## Discussion

IgG4-RD may affect multiple organs in 60 to 90% of patients with IgG4-RD, including cardiovascular organs [3–5]; it responds positively to corticosteroid therapy [1]. Previous studies have suggested that CP could often develop after mediastinal or idiopathic retroperitoneal fibrosis due to IgG4-RD [5] and pleural and/or cardiac effusion was sometimes observed continuously [6]. However, few reports have assessed the effusion by cytological examination with immunostaining. Diagnostic criteria for IgG4-RD have been proposed as follows [2]: (1) typical tissue fibrosclerosis; (2) elevated serum IgG4 (>135 mg/dL); and (3) histopathological features, including lymphocyte infiltration and a high ratio of IgG4-positive plasma cells/IgG-positive plasma cells (>40%). In our case, our patient’s serum IgG4 level did not exceed the reference value; however, IgG4-positive lymphocytes in his cardiac effusion led us to perform further examinations of IgG4-RD. Because the findings of the cytological examination did not meet diagnostic criterion (3) properly, we performed an ^18^F-FDG-PET scanning to judge the extent of inflammatory foci [7–10]. The ^18^F-FDG-PET scanning showed abnormal uptake in atypical organs such as his pericardium, his gastric wall, and in his hilar lymph nodes, but not in his pancreas, his lacrimal and salivary glands, or kidneys that were reported as common sites of IgG4-RD [3]. Finally, we performed a thoracoscopic pericardiectomy because of ^18^F-FDG-PET findings.

Only a few studies have reported cytological examinations in patients with IgG4-RD. Kabara *et al*. [11] demonstrated a case of IgG4-RD in which IgG4-positive plasma cells were detected in the pericardial effusion by fine-needle aspiration cytology [11]. They diagnosed the case as having IgG4-RD and prescribed prednisolone; however, gallium scintigraphy showed no abnormal uptake and a histopathological evaluation was not performed. Therefore, they did not indicate the association between inflammatory foci and IgG4-related plasma cells in the pericardium. The possible reason for insufficient proof of inflammation was that gallium scintigraphy might have a low signal-to-noise ratio in diagnostic imaging of IgG4-RD [12]. On the other hand, a previous report demonstrated that ^18^F-FDG-PET revealed hypermetabolic lesions in 97.1% patients with IgG4-RD [13] and was a superior imaging modality for demonstrating the extent, compared to ^67^gallium scintigraphy [12]. Our case report suggested that ^18^F-FDG-PET scanning should be chosen to make a definitive histopathological diagnosis after identifying the presence of IgG4-RD by a cytological evaluation.

## Conclusion

We report the rare case of a patient with IgG4-RD who presented with CP that was identified by a cytological examination in pericardial effusion and diagnosed by a histopathological analysis of the pericardium.

## Abbreviations

^18^F-FDG: ^18^F-fluorodeoxyglucose
CP: Constrictive pericarditis
IgG: Immunoglobulin G
IgG4: Immunoglobulin G4
IgG4-RD: Immunoglobulin G4-related disease
PET: Positron-emission tomography
TTE: Transthoracic echocardiography
